# MG-63 osteoblast-like cell proliferation on auxetic PLGA scaffold with mechanical stimulation for bone tissue regeneration

**DOI:** 10.1186/s40824-016-0080-4

**Published:** 2016-10-31

**Authors:** Hong Jin Choi, Jun Jae Lee, Yeong Jun Park, Jung-Woog Shin, Hak-Joon Sung, Ji Won Shin, Yanru Wu, Jeong Koo Kim

**Affiliations:** 1 Department of Biomedical Engineering, Inje University, Obang-Dong, Gimhae, Gyeongnam 621-749 Korea; 2 Department of Biomedical Engineering, Vanderbilt University, Nashville, TN 37212 USA

**Keywords:** Negative Poisson’s ratio, MG-63 cell, Mechanical stimulation

## Abstract

**Background:**

Auxetic scaffolds (experimental) was fabricated by using poly(D, L-lactic-co-glycolic acid), 50:50, (PLGA) for effective bone cell proliferation with mechanical stimulation.

**Methods:**

Negative Poisson’s ratio in scaffold, 3-directional volumetric compression was applied during the scaffold fabrication at adequate temperature (60 °C). The pore size of scaffold ranged between 355 and 400 μm.

**Results:**

The porous morphology of the prepared auxetic scaffolds had shown partially concave and dent shapes in SEM image as expected. The lowest Poisson’s ratios of experimental group was −0.07 at 60 °C/10 min. Compressive strength of experimental group was shown about 3.12 times higher than control group (conventional scaffold) in dry state at 25 °C. The compressive strengths of both groups were tended to be decreased dramatically in wet state compared to in dry state. However, compressive strengths of experimental group were higher 3.08 times and 1.88 times in EtOH/PBS (25 °C) and EtOH/PBS/DMEM (37 °C) than control group in wet state, respectively. Degradation rate of the scaffolds showed about 16 % weight loss in 5 weeks. In cell attachment test, experimental group showed 1.46 times higher cell proliferation than control group at 1-day with compressive stimulation. In 3-day culture, the experimental group showed 1.32 times higher than control group. However, there was no significant difference in cell proliferation in 5-day cultivation.

**Conclusion:**

Overall, negative Poisson’s ratio scaffolds with static mechanical stimulation could affect the cell proliferation at initial cultivation time.

## Background

Recently, biodegradable polymer scaffolds have been utilized in tissue engineering to regenerate or substitute for a human organs [1, 2]. So numerous researchers had studied and utilized the 3-D scaffolds via various methods and aspects such as using various pore size [2]. The results in many papers are emphasized and come out with numerous characteristics of the 3-D scaffold such as the pore size, porosity, and the mechanical property for tissue engineering [2–4].

Thus, the characteristics of the 3-D scaffold such as mechanical properties, pore size, surface property, and degradability were very important as a culture media for the tissue engineering. The characteristics of the scaffold is also depends on the cell or tissue types. For example, the pore size was also employed differently such as 380~405 μm pore size for chondrocyte and osteoblast growth, and 186~200 μm pore size for fibroblast growth [3]. Besides, there are also many researchers had studied mechanical properties and degradation behaviors for the various biodegradable polymer scaffolds [1, 4–6]. The mechanical properties and degradation behaviors are among the important factors because they are related to the structural and mechanical stability for the tissue regeneration [1, 5]. Thus, the physical properties of 3-D scaffolds must be taken into consideration by using innovative approaches. One of the most important factors for cell proliferation is mechanical stimuli to the cell. There are various methods for mechanical stimulation that will affect the cell behaviors such as compressive loading that is divided into hydrostatic pressure and platen displacement in a 3-D specimen, longitudinal stretch, substrate bending, plane substrate distention, and fluid shear system [7].

Based on Wolff’s law, the bone cell tends to proliferate by the compressive stimulation because bone composition has piezoelectric properties and bone remodeling is caused by various stimulations [8]. In addition, Compressive stimulation can lead to osteogenic gene expression for proliferation and differentiation [9, 10]. It would depends on the Poisson’s ratio in order to determine the compressive stimulation acted on the bone cells in 3-D scaffolds. The conventional 3-D scaffolds or matrices including all common materials have a positive Poisson’s ratio [11].

The Poisson’s ratio is defined as the negative strain ratio of the longitudinal strain divided by the transverse strain [11, 12]. If the material has a negative Poisson’s ratio, it will conduct high compressibility in multi axial directions, and the material is known as auxetic material [13]. Therefore, the 3-D scaffold with negative Poisson’s ratio will be expected undergoing the bone cell proliferation effectively.

Lakes proposed the methods to form a negative Poisson’s ratio to the foam structures, that is, a permanent volumetric compression in three orthogonal directions and heat processing [12]. Lakes and Choi formed the polyurethane foam with a negative Poisson’s ratio via volumetric compression ratio and heat processing [13]. The cell size of foam, different heating temperature and heating time are affected to the value of negative Poisson’s ratios [14]. There are several important conditions such as pore size, compression ratio, heating temperature and heating time needed to form a negative Poisson’s ratio. Kim formed the polyurethane foam with a negative Poisson’s ratio and utilized it as the scaffolds for cartilage regeneration via mechanical stimulation [15].

This study focuses on fabrication of auxetic PLGA scaffolds and investigate its effectiveness of the bone cell proliferation with compressive stimulation.

## Methods

poly((D,L-lactic-co-glycolic acid) (PLGA, Lakeshore Biomaterials, Essen, Germany) was used for scaffold fabrication. The composition ratio of lactic and glycolic acid (LA : GA molar ratio) is 50:50. Molecular weight (Mw, Mn) was 124 kDa and 77 kDa and the glass transition temperature (Tg) was 47.7 °C. For scaffold fabrication, chloroform was employed as a solvent and sodium chloride particles were used as porogen in the scaffold.

### Fabrication of conventional scaffold vs. auxetic scaffold

Solvent casting/salt leaching methods were used to fabricate PLGA porous scaffold. Sodium chloride particulates in the range of 355~400 μm particle size were used for control group and 500~600 μm for experimental group. Both groups were prepared by using a Teflon mold. Control specimens were prepared as 1.5 × 1.5 × 1.5 (cm^3^) in size and experimental specimens were prepared as 2 × 2 × 2 (cm^3^) and 2.2 × 2.2 × 2.2 (cm^3^) in size. In order to make same pore size after applying compression to the experimental specimens, the pore size was bigger for experimental specimen.

PLGA copolymer was dissolved in chloroform and then mixed with sodium chloride particles. The mixture solution was placed in the mold and then dried at room temperature for 1 day. After drying, the mixture was removed from the mold and washed with distilled water for 2 days in order to remove sodium chloride particles. Finally, specimens were freeze-dried for 24 h.

**Fig. 1:**
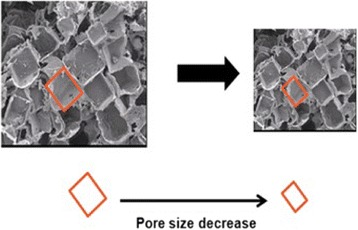
Make same pore size to control specimens via permanent volumetric compression. Volumetric compression ratios are 2.4 and 3.1. 2 × 2 × 2 (cm3) to 1.5 × 1.5 × 1.5 (cm3) size is 2.4 : 1 and 2.2 × 2.2 × 2.2 (cm3) to 1.5 × 1.5 × 1.5 (cm3) size 3.1 : 1

Permanent volumetric compression with heat treatment (PVCT) method was employed in order to create a negative Poisson’s ratio to the scaffolds [9]. For the experimental specimen, heat treatment was applied to the temperature above its Tg in a drying oven (DMC-122, DJDAEIL, Daejeon, Korea). The specimen was then compressed in three orthogonal directions at the same time. Firstly, heat treatment was applied to the specimens at 50 °C/5 min, 50 °C/10 min, 60 °C/5 min and 60 °C/10 min. Secondly, tri-axial compression was applied with 2.4:1 and 3.1:1 in volumetric ratio. The experimental specimens were compressed in three orthogonal directions by two Teflon molds up to 1.5 × 1.5 × 1.5 (cm^3^) in size and became the same size as that of control (see Fig. 1). The specimens were then cooled at room temperature and removed from the mold.

### Analysis of morphology for SEM

The surface topography of the freeze dried PLGA scaffolds were examined by a scanning electron microscope (SEM, HITACHI 2-2400). All scaffolds were cut half a scaffold, were checked a cross section by SEM.

### Measurement of Poisson’s ratio

**Fig. 2:**
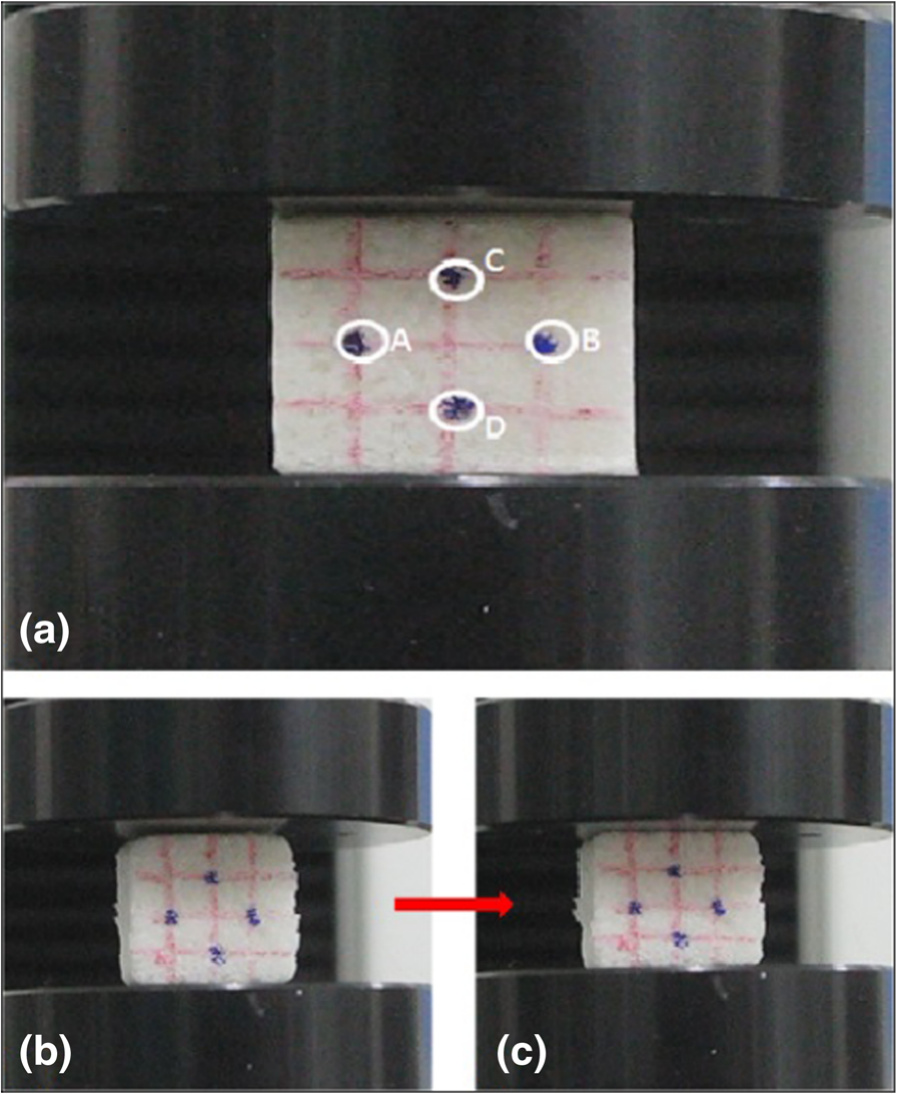
Points were tracked to estimate Poisson’s ratio of the specimen by image processing. (**a**). (**b**) to (**c**) is changing image when the specimen was compressed

The Poisson’s ratio for all specimens was analyzed by image processing with Image J program v2.0.0. The Material Testing System (LRX-PLUS, Lloyd instruments, West Sussex, UK) was utilized to test the specimens. A digital microscope (BX51, Olympus Corporation, Tokyo, Japan) was used to capture images in order to measure the displacement of the specimen (see Fig. 2).

1 $$ {\varepsilon}_{\mathrm{x}}=\frac{\left|A-B\right|-\left|A0-B0\right|}{\left|A0-B0\right|} $$

2 $$ {\varepsilon}_{\mathrm{y}}=\frac{\left|C-D\right|-\left|C0-D0\right|}{\left|C0-D0\right|} $$

3 $$ \nu = \frac{\upvarepsilon \mathrm{x}}{\ \upvarepsilon \mathrm{y}} $$

where:

ε_x_: strain of x-axis.
ε_y_: strain of y-axis.
ν: material Poisson’s ratio.
A0~D0: initial point (load = 0).
A~D: moved point under load.

Where ε_x_ is the strain of x-axis, ε_y_ is the strain of y-axis and ν is Poisson’s ratio. A_0_ to D_0_ are initial points (0 % strain) and A to D are changed points under applying a load.

All specimens were compressed from 0 to 25 % strain and the image was captured with 5 % strain intervals. The points A to D were utilized to calculate the Poisson’s ratio by tracking the center of point as shown in Fig. 2. The changes of x-axis and y-axis distance were calculated by using Eqs. (1) and (2), thereby Poisson’s ratio was calculated by Eq. (3).

### Mechanical properties of the scaffold

The compressive strengths of the control and experimental groups were analyzed under the conditions shown in Table 1 by using a Material Testing System (MTS) at 10 % compressive strain. The cross head speed of MTS was 1 mm/min.

**Table 1:** Conditions of compressive strength measurement for the scaffolds in dry and wet state

Types	Conditions	Temperature
A	dry/25 °vC (experimental)	25 °C
B	dry/25 °C (control)	25 °C
C	wet/EtOH/PBS (experimental)	25 °C
D	wet/EtOH/PBS (control)	25 °C
E	wet/EtOH/PBS/DMEM (experimental)	37 °C
F	wet/EtOH/PBS/DMEM (control)	37 °C

### Degradation of the scaffold

In order to analyze the degradation characteristics of the scaffold, we prepared a disc-shaped scaffold. (Diameter = 12 mm, height = 2 mm) The fabricated scaffold was pre-wetted in 70 % ethanol, and then placed in each of 4 ml phosphate buffered saline solution (PBS, pH 7.4). The change in weight of the scaffold in a shaking incubator at 37 °C for 7 weeks was measured. The degradation rate of the scaffold was calculated by Eq. (4).

4 $$ \mathrm{Degradation}\kern0.5em \mathrm{rate}\left(\%\right)=\frac{Initial\kern0.5em  weight- loss\kern0.5em  weight}{Initial\kern0.5em  weight}\times 100 $$

### Cultivation of MG-63 cells

Human MG-63 osteoblast-like cells (KCLB, Seoul, Korea) were used for cell attachment and proliferation test. They were cultured at 37 °C in a humidified 5 % CO_2_ and cultured in DMEM containing 10 % fetal bovine serum and 1 % penicillin/streptomycin. Cells were sub-cultured every 3 days at 90 % confluence in 75 cm^2^ and 25 cm^2^ culture flask. 4~6 passage cells were used for this study. The cellular experiments were performed in 24-well plate and 12-well plate where each well was immersed by the culture medium.

### Analysis of initial cell attachment for the scaffold

In cell attachment test, the MG-63 osteoblast-like cells were seeded in density of 3.6 × 10^5^ cells/40 μl for each specimen. After cell seeding, they were incubated for 2 h and then added in immersed culture medium. At each time point (4, 8, 12 and 24 h), specimens were moved to new 24-well plates and washed two times with PBS solution. The attachment rate was measured by using cell counting kit-8 (CCK-8, Sigma-Aldrich) by absorbing read on a micro-plate reader at 450 nm.

### CCK-8 test for cell proliferation by static compressive stimulation

**Fig. 3:**
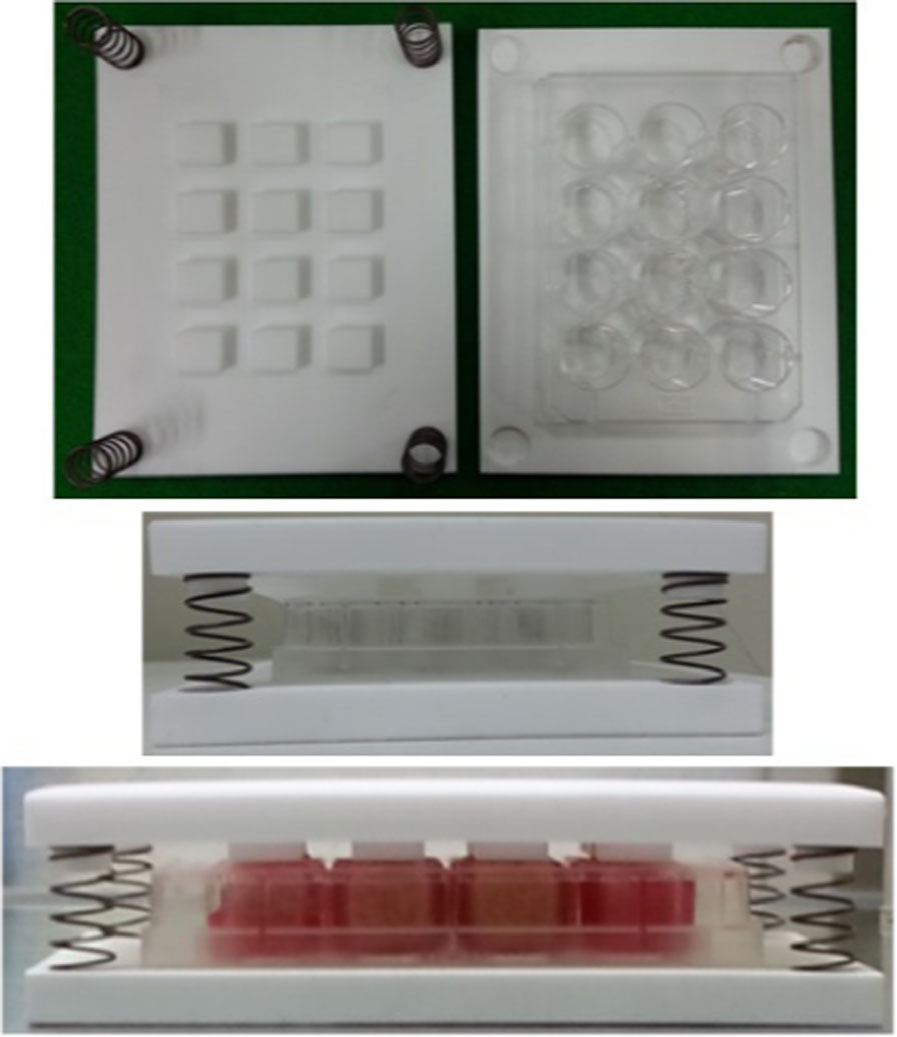
An apparatus for cell culture with compressive stimulation. The apparatus was manufactured to apply 10 % compressive strain to the scaffold in 12-well plate

We prepared our own compressive stimulation device as shown in Fig. 3. It can control applying load with 10 % strain elongation to the scaffold. The applied load was 19.6 Newton. The cells (1.8 × 10^5^ cells/80 μl) were seeded onto the control and experimental specimen groups. The compressive stimulation was applied after 1 day from cell seeding to prevent the loss of cells from the specimens. The cells were seeded into the inner pore of specimens to prevent the damage of cells by compression.

Cell proliferation test were divided into three types as shown in Table 2. The cells were seeded onto the specimens at a density of 1.8 × 10^5^ cells/80 μl. After the cells’ attaching to the specimens, the compressive stimulation was applied to the cells by using the prepared apparatus. At each time of 1-, 3- and 5-day culture, the specimens were moved to new 12-well plates and added with fresh culture medium. Ten percent (*v/v*) CCK-8 reagent was added to immerse well and incubated at 37 °C in a humidified 5 % CO_2_ for 4 h. One-hundred microlitre of the reactant were moved into a 96-well plate and the absorbance read was obtained on a micro-plate reader at 450 nm. The cell number was calculated by initial absorbance value.

**Table 2:** Conditions of cell proliferation experiment in accordance with the stimulation

Scaffold types	Compressive stimulation	Conditions
Control group	X	37 °C/5 % CO_2_
Control group	O	37 °C/5 % CO_2_
Experimental group	O	37 °C/5 % CO_2_

### Statistical analysis

Data were expressed as mean ± standard error for all comparisons. A *t*-test was used to evaluate differences between groups (p <0.05).

## Results and discussion

### Morphology of scaffolds

**Fig. 4:**
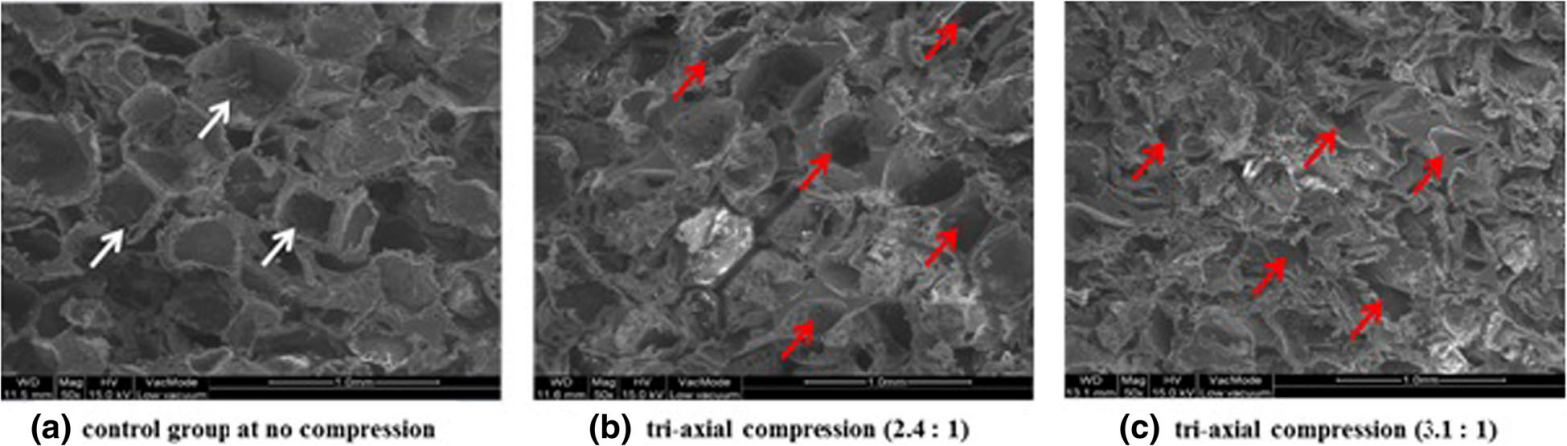
Cross-sectional SEM images of PLGA scaffold. **a** control specimen before compression. **b** experimental specimen after tri-axial compression (2.4 : 1), (**c**) experimental specimen after tri-axial compression (3.1 : 1). The *arrows* indicate difference of pore structure

Structure of negative Poisson’s ratio was obtained successfully from the process of heat treatment with 3-D compression for the scaffold. Concave porous structure was created in the scaffold specimens. The cross-sectional microstructure of the PLGA scaffold is shown in Fig. 4. The porous shape of control specimen was observed as a cubical and convex type as shown in Fig. 4(a). In contrast, experimental specimen showed partially concave and dented shapes as shown in Fig. 4(b) and (c). This transformation of porous shape is resulted from changing micro porous structure of the scaffold by tri-axial compression. The porous structure of these recessed and dented shapes enable the scaffold have negative Poisson ratio.

### Poisson’s ratio of scaffolds

**Fig. 5:**
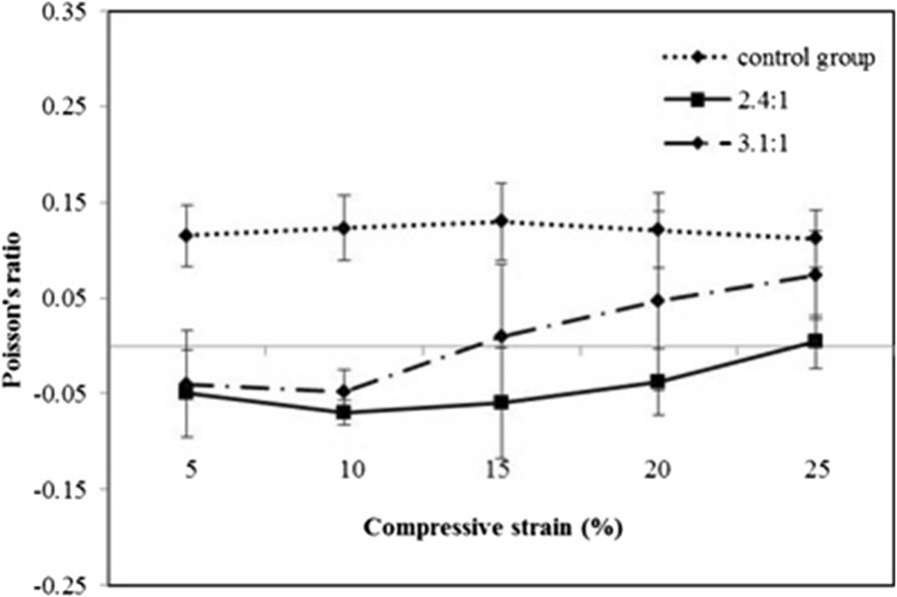
Poisson’s ratio variations for control and experimental specimens. The volumetric compression ratio was 2.4 and 3.1 (*n* = 4, *p < 0.05)

The Poisson’s ratio of a control specimen was shown as about 0.11~0.13 when compressed at 5 to 25 % strain (see Fig. 5). For the experimental specimen, the Poisson’s ratios in accordance with the permanent volumetric compression with heat treatment (PVCT) ratio of 2.4 and 3.1 were shown as −0.07 and −0.05, respectively. The specimen of PVCT ratio of 2.4 showed more negative Poisson’s ratio than that of PVCT ratio of 3.1. Thus, extremely high compression ratio of the PVCT ratio of 3.1 can be considered as a factor to cause the shape of the pore structure to collapse. Therefore, PVCT ratio of 2.4 is considered as more effective ratio to form negative Poisson’s ratio.

**Fig. 6:**
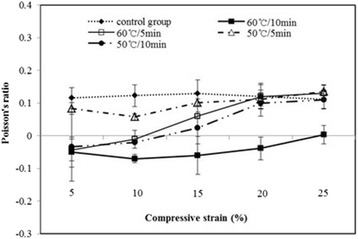
Poisson’s ratio variations for control and experimental specimens. Processing was done at 50, 60 °C and for 5, 10 min with a volumetric compression ratio of 2.4 (*n* = 4, *p < 0.05)

The heat treatment temperatures (50, 60 °C) and heating time (5, 10 min) with a PVCT ratio of 2.4 were applied during the forming process of a negative Poisson’s ratio. Poisson’s ratios of experimental group were 0.05 at 50 °C/5 min, −0.03 at 50 °C/10 min, −0.04 at 60 °C/5 min and −0.07 at 60 °C/10 min as shown in Fig. 6, respectively. The result showed that PLGA50:50 scaffolds specimen depended on heating temperature and heating time to reveal negative Poisson’s ratio. The lowest negative Poisson’s ratio was −0.07 with 60 °C/10 min at 10 % strain (see Fig. 6). These results are important because the different heating temperature and heating time for the PVCT ratio would affect the negative Poisson’s ratio.

### Mechanical properties of the scaffold

**Fig. 7:**
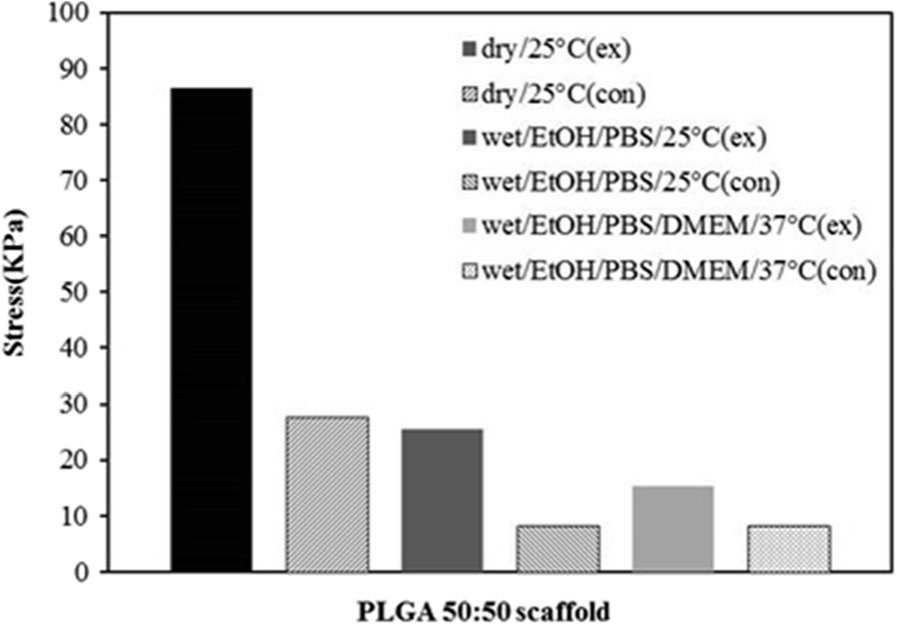
Compressive strengths for the scaffold in dry and wet state

Compressive strengths of the scaffolds in dry and wet state were shown at Fig. 7. Compressive strength of experimental group was about 3.12 times higher than the control group in dry state at 25 °C. The compressive strengths of all groups were tended to be decreased dramatically in wet state than those in dry state. However, compressive strengths of experimental group were higher 3.08 times and 1.88 times at EtOH/PBS (25 °C) and EtOH/PBS/DMEM (37 °C) solution than the control group, respectively. Therefore, the experimental specimens are considered to be able to compensate for decrement of the mechanical strength of the control group in wet state. NPR scaffolds were improve the strength in wet environment compared with control scaffolds.

### Degradation of the scaffold

**Fig. 8:**
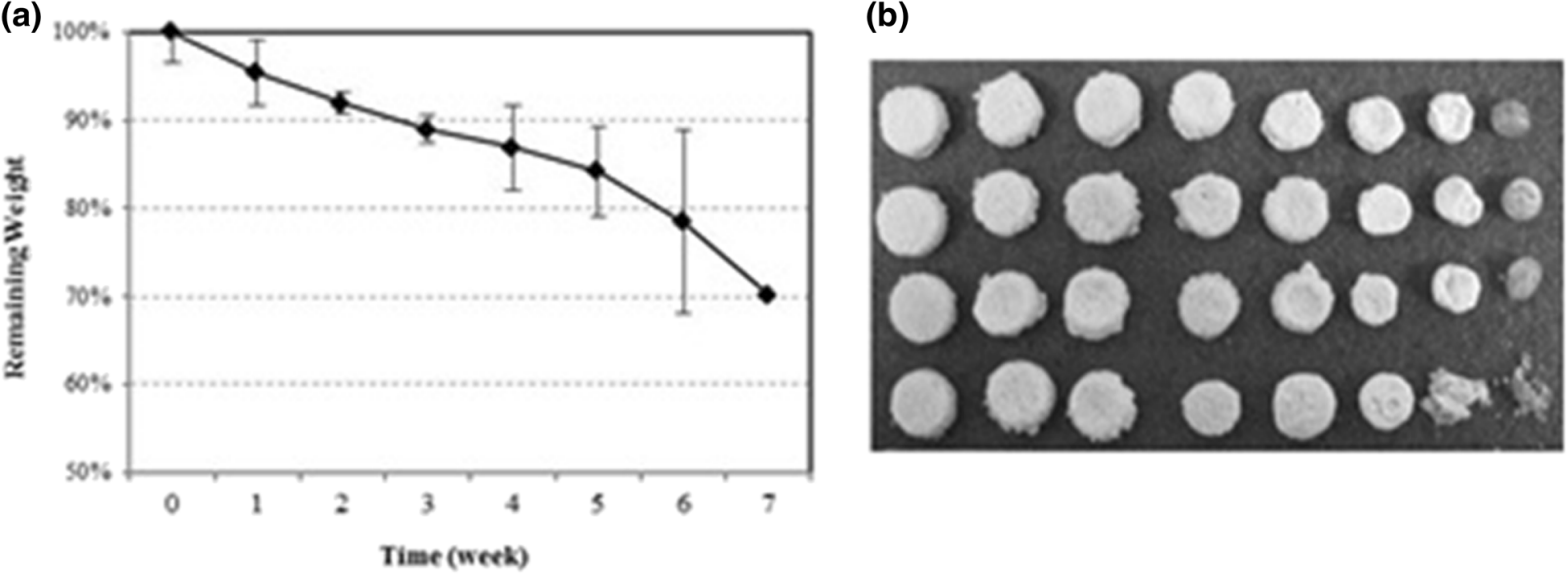
Degradation of control specimens in PBS solution during 7 weeks (**a**) Degradation rate and (**b**) morphology change

Degradation rates and morphology changes of the PLGA scaffolds in PBS solution during 7 weeks were shown in Fig. 8. As time passed, the weight of the scaffold had been reduced (see Fig. 8(a)). The degradation rate of the scaffold is shown about 16 % weight loss after 5 weeks, but morphology of the scaffold was maintained. The collapse of the scaffold occurred after 6 weeks passed (see Fig. 8(b)).

### CCK-8 test for cell proliferation by static compressive stimulation

**Fig. 9:**
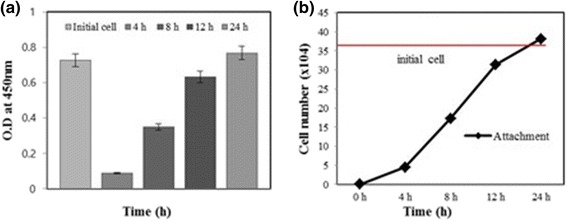
MG-63 cell attachment rate for 1 day for control and experimental specimens. Optical density at 450 nm (**a**) and cell number (**b**) of cell attachment at each time point (*n* = 4, *p < 0.05)

In cell attachment test, the cell attachment at time points of 4, 8, 12 and 24 h were 12.5, 50.0, 86.1 and 106.0 %, respectively, as shown in Fig. 9. This result means that MG-63 cells needed at least 24 h in order to attach inside of the PLGA scaffold. Because of applying compressive stimulation on scaffold, the seeded cells should be placed inside of the scaffolds as well as enough number of cells should be attached in/on scaffolds.

**Fig. 10:**
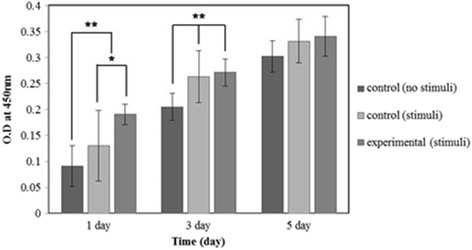
MG-63 cell proliferation rate on PLGA scaffolds at 1, 3 and 5 day culture with static stimulation (*n* = 4, *p < 0.05, **p < 0.01)

The results of cell proliferation with and without stimulation on control and experimental scaffold specimens are shown in Fig. 10. At 1-day and 3-day culture, there is significant difference between with and without compressive stimulation. Additionally, the experimental group showed 1.46 times higher cell proliferation than control group at day-1 with compressive stimulation, and the experimental group showed 2.09 times higher cell proliferation than control group at 1-day without compressive stimulation. In case of 3-day culture, the experimental group showed 1.32 times higher than control group with no compressive stimulation. However, when applying a compressive stimulation, there was no significant difference between experimental group and control group at 3-day culture. At 5-day culture, there is no significant difference between all groups.

As a result of cell proliferation test, the effectiveness of negative Poisson’s ratio was revealed at 1-day and 3-day culture. However, there was no significant difference in cell proliferation due to diminish of mechanical strength of the scaffold as well as disappear the negative Poisson’s ratio in cultivation at 5-day. The diminishment is presumably caused by viscoelastic behavior of the PLGA in the liquid culture media. Hence, static compressive stimulation is no more effective in 5-day culture period due to mechanical strength reduction and stress relaxation in the scaffold. This might be mechanical stimulation to the scaffold could not be sufficiently transmitted to cells.

Overall, the static mechanical stimulation on auxetic PLGA scaffold would affect the cell proliferation in short period. Presumably, dynamic stimulation might be more effective on cell proliferation than the static stimulation during cultivation [16].

## Conclusions

A negative Poisson’s ratio was moderately formed for the PLGA scaffolds. As previously mentioned, there were various factors to form a negative Poisson’s ratio for foam structure such as tri-axial compression, heat treatment temperatures and time. In this study, negative Poisson’s ratio were successfully embodied, and auxetic PLGA scaffold could be fabricated with the permanent volumetric compression ratio of 2.4 and 60 °C/10 min conditions. The MG-63 osteoblast-like cells were entirely attached after 24 h on the scaffold. The bone cells were grown well via the compressive stimulation. From 1-day to 3-day culture, the result showed the effectiveness of negative Poisson’s ratio of the scaffold for bone cell proliferation. It is considered that the auxetic PLGA scaffolds would affect initial period for bone cell proliferation effectively. Hence, we conclude that mechanical stimulation for bone cell cultivation through auxetic scaffolds should have had beneficial aspects and might be play an important role in bone tissue regeneration.

## Abbreviations

SEM: Scanning electron microscope
PLGA: poly(D, L-lactic-co-glycolic acid)
PVCT: Permanent volumetric compression with heat treatment
MTS: Material Testing System
PBS: Phosphate buffered saline solution
DMEM: Dulbecco’s modified Eagle’s medium
CCK-8: cell, counting kit-8
NPR: Negative Poisson ratio
